# The Feasibility and Acceptability of an Online CPD Programme to Enhance PE Teachers’ Knowledge of Muscular Fitness Activity

**DOI:** 10.3390/ijerph191912132

**Published:** 2022-09-25

**Authors:** Ashley Cox, Robert J. Noonan, Stuart J. Fairclough

**Affiliations:** 1 Movement Behaviours, Health, and Wellbeing Research Group, Department of Sport and Physical Activity, Edge Hill University, Ormskirk L39 4QP, UK; 2 Appetite and Obesity Research Group, Department of Psychology, University of Liverpool, Liverpool L1 8JX, UK; 3 Faculty of Health and Wellbeing, University of Bolton, Bolton BL3 5AB, UK

**Keywords:** muscular fitness, physical activity, adolescents, physical education, continued professional development

## Abstract

Schools provide an opportunity to enhance muscular fitness of English youth during physical education (PE). Continued professional development (CPD) among teachers may improve muscular fitness activity delivery in schools. This study sought to assess the feasibility and acceptability of an online CPD programme to enhance PE teachers’ knowledge of muscular fitness activity. Methods: Co-production of an online CPD platform was undertaken with five secondary school PE teachers. A quasi-experimental pre-post control group design for the CPD was adopted (CPD group *n* = 76, control *n* = 32). Upon CPD completion feedback was solicited for qualitative analysis. Results: Pre-and-post CPD knowledge quiz data were available from 65 participants (55.4% male). The median knowledge quiz change score was significantly higher in the CPD group than in the wait list control group (CPD score vs. control score; U =37, z = −5.96, *p* < 0.01). Three primary themes reflecting factors associated with the acceptability and feasibility were (1) practical application, (2) support and resources, and (3) knowledge and confidence. Conclusions: Co-production of an online CPD programme can improve knowledge and affect practice. PE teachers that completed the CPD reported the online platform was beneficial to overcoming the limitations of face-to-face CPD such as time and financial constraints and suggested the content covered was beneficial and appropriate to their teaching. Future work is required to establish links between teachers’ learning following CPD, the translation into PE practice and student MF outcomes.

## 1. Introduction

Physical activity (PA) guidelines for the UK and other developed countries recommend children and young people (referred to as youth from here) engage in a minimum of 60 min of moderate-to-vigorous intensity PA (MVPA) per day, averaged across the week with three days to incorporate muscle and bone strengthening exercise to develop movement skills, bone strength, and muscular fitness (MF) [1,2]. MF activity provides a range of health benefits including improvements in metabolic function, bone health, and mental health [3,4]. Furthermore, MF is associated with enhanced fundamental movement skills, reductions in injury rates, and increased MVPA [5,6,7]. Meeting the recommended amount of MF activity in youth can reduce the likelihood of adverse health outcomes in adulthood and prepare youth for a lifetime of PA participation [8]. 

Schools have the facilities and the physical education (PE) curricula to promote and support health promotion including fitness programmes independent of a pupils’ sociodemographic profile [9,10]. As such, schools are suitable settings to promote and support MF activity [11,12,13,14,15,16]. Notwithstanding this, MF activity seldom features within PE curricular due to perceived barriers among some PE teachers. These barriers tend to centre around factors such as limited confidence and time and perceived lack of equipment and facilities [17,18,19]. Moreover, research from the US suggests that PE teachers’ knowledge required to deliver safe and appropriate MF activity in schools requires improvement [20]. Although the literature surrounding MF activity delivery in UK schools is limited, there is evidence of successful interventions to improve knowledge of MF activity delivery elsewhere. Kennedy and colleagues delivered one-day face-to-face continued professional development (CPD) workshops comprising of theoretical and practical MF activity content to school teachers across 16 schools in Australia [21,22]. They found that one-day face-to-face workshops increased teachers’ confidence in delivering MF activity to adolescents. This study highlighted that PE teachers’ knowledge of MF activity can be improved and their perceived barriers overcome through participating in CPD. However, CPD opportunities tend to be limited in the UK and there is currently a lack of understanding as to the efficacy of PE CPD programmes in the UK.

Traditionally, PE teacher CPD has been constrained by the time and financial demands associated with face-to-face training [23]. However, these time and financial barriers have been overcome in recent years with the opportunity to deliver training remotely via online platforms [24]. However, online CPD provision for PE teachers, particularly in the UK is in its infancy, and further research is warranted to better understand the feasibility and acceptability of online CPD delivery to UK PE teachers [25]. Of the few studies that have been conducted in this area [25,26,27,28], there is some evidence to suggest that PE teachers favour online CPD that: (1) evidence-based; (2) provides pedagogical content knowledge and not just content knowledge; (3) is informed by teachers and translatable to practice; (4) facilitates communities of practice; (5) interactive; (6) simple to navigate; and (7) highly visual [25]. We sought to build on these findings in the present study. The overarching aims of the present study were to (1) assess if an online CPD course can increase PE teacher knowledge of MF activity (2) assess the feasibility of an online CPD course to enhance PE teachers’ knowledge and competence of MF activity, and (3) assess the acceptability of the content and design of an online CPD course from PE teachers’ perspectives.

## 2. Materials and Methods

### 2.1. CPD Conceptualisation

CPD conceptualisation followed the distribution of an online survey to secondary school PE teachers investigating PE teachers’ perceived expertise and CPD requirements in the delivery of MF activity. The survey comprised of 21 questions organised into four sections that gathered information about: (1) the respondents and their schools, (2) their policies and practices with regard to the promotion of PA and MF, and their views and perceptions of these, (3) their professional training, and finally (4) their suggestions and thoughts regarding MF. The findings of the survey are currently under review elsewhere. However, it was identified there was no MF CPD undertaken by the 194 respondents from across the UK. Surveyed teachers delivering PE from across the UK believed that their knowledge of school-based MF activity required development, and this lack of knowledge was reflected in a limited understanding of programme design and the benefits of MF activity. The preliminary findings of this survey are supported by previous work conducted in the US, whereby PE teachers required training specific to the design and implementation of MF activity in secondary schools [20]. Therefore, initial CPD design was undertaken by the lead author with a focus on developing foundational knowledge of MF, with a focus on the health benefits and delivery of MF. The authors all have experience as lecturers in higher education and are experienced in the development and delivery of educational content. Four recent publications that provided guidance on how best to deliver MF activity in school settings were used to identify broad topic areas to be covered within the CPD programme [12,13,29,30]. This approach to CPD development ensured that the programme content was evidenced based which is considered essential to successful CPD design [25]. Furthermore, secondary schools were deemed an appropriate setting for the CPD programme given the possibility of effective implementation as reported in recent literature [11,13]. The main topic areas covered within the CPD programme were: PA, MF development, plyometrics, delivery and long term development and monitoring and assessment.

### 2.2. CPD Development

A user-centred approach [31] to the CPD design was undertaken with five secondary school PE teachers who had participated in previous research led by the first author. The user-centred approach allowed for an iterative design process that involved PE teachers in the design of their own CPD to improve implementation fidelity [31]. The teachers engaged in a collective process that led to establishing a consensus on the CPD content and knowledge assessment. This approach to CPD development ensured that the content was informed by the needs of the teachers which is associated with improved CPD engagement [25]. Three face-to-face meetings with the first author and the five PE teachers were conducted. Three of the teachers joined the first author in person with the remaining two teachers joining via zoom (mean duration: 48 ± 7 min). This approach to PE teacher CPD and examination design is similar to that previously reported in the US [20]. Brief summaries of each meeting are provided below.

#### 2.2.1. Meeting One

Meeting one consisted of reviewing the survey distributed amongst UK PE teachers to assess perceived expertise and CPD requirements for the delivery of MF activity. Open ended queries to solicit recommendations for CPD content were created and these formed indicative CPD content. The initial CPD content identified by the lead author was proposed to the PE teachers as a potential framework. The PE teachers agreed that the proposed framework would meet their needs, and further emphasised the importance of CPD content being evidence informed. Following the meeting, the first author developed a CPD content plan, expanding on the points raised in meeting one to propose at the next meeting. 

#### 2.2.2. Meeting Two

Meeting two occurred four weeks after meeting one. It involved developing the online CPD learning modules and agreeing on CPD content and delivery. CPD was developed and delivered via Talent LMS (Version 4.3), a cloud-based platform which uses open-source code and is publicly available (TalentLMS.com). Talent LMS was selected as the host platform as it provides the flexibility to use a range of media including PowerPoint presentations, video, built in quizzes and discussion forums [32]. Moreover, the level of flexibility provided by the platform allowed for the CPD content and delivery to be interactive, highly visual and easy to navigate which are important characteristics of effective PE teacher CPD [25]. Information and suggestions provided by the PE teachers were aligned with existing recommendations for online CPD development for PE teachers [25]. This ensured that the CPD (1) was evidence-based, (2) provided pedagogical content knowledge and not just content knowledge, (3) was informed by teachers and translatable to practice, (4) facilitated communities of practice, (5) was interactive, (6) was simple to navigate, and (7) was highly visual [25].

#### 2.2.3. Meeting Three

Meeting three occurred five weeks after meeting two. It allowed PE teachers to review preliminary MF activity knowledge assessment questions proposed by the first author in the form of an online quiz. PE teachers offered suggestions regarding the quiz questions' wording, correct answers, and distractors. Distractors were intended to distinguish between PE teachers who have not yet acquired the knowledge necessary to answer the item correctly from those who understand the content. Therefore, the distractors in the multiple-choice questions were designed to contain plausible but incorrect answers based on teachers’ common errors or misconceptions of MF in order to measure teachers’ level of knowledge acquisition [33].

Following meeting three, the PE teachers reviewed the CPD platform, content, and knowledge quiz content. Once the teachers were satisfied with the platform’s feasibility and useability, it was deemed ready for use. A summary of CPD modules can be seen in Table 1. Modules were linearly ordered to build knowledge as the teachers worked through the CPD programme. During the design and development process, the PE teachers suggested that professionally recognised CPD points would provide an incentive to complete the CPD programme. Endorsement of CPD points was subsequently sought from the British Association of Sports and Exercise Sciences and approved for five CPD credits on 21 October 2021.

### 2.3. CPD Participant Recruitment and Distribution

**Figure 1:**
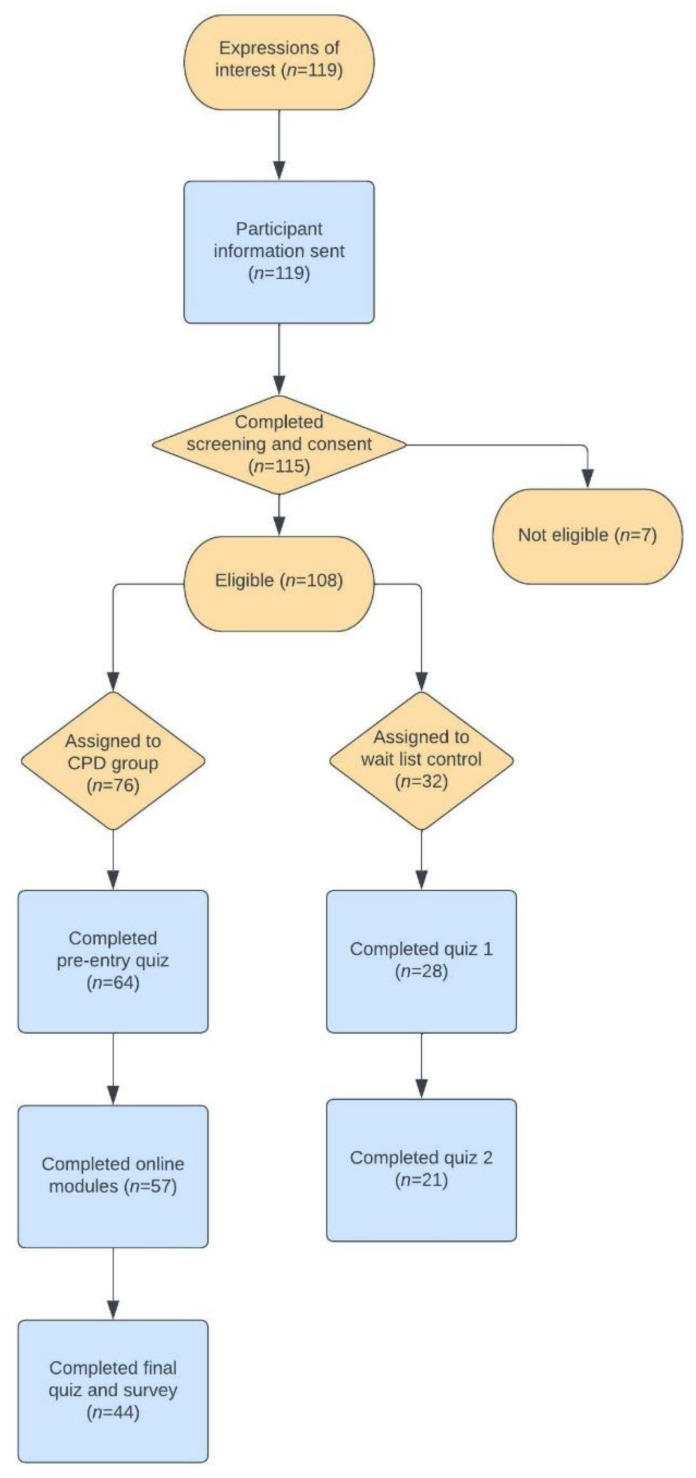
Participant recruitment breakdown.

Data collection took place between November 2021 and January 2022. A combination of convenience and purposeful sampling was used to recruit secondary school PE teachers from the UK to participate in the CPD. A study recruitment message detailing the nature of the study, eligibility criteria, and a link to the CPD was provided to all potential participants following the distribution of an online survey. Teachers were eligible to participate if they were current UK secondary school PE teachers. One hundred and nineteen teachers expressed an interest in taking part. A screening process was undertaken via the CPD platform to ensure that potential participants met the eligibility criteria. One hundred and fifteen PE teachers completed the screening process and provided consent. Seven teachers were identified as not having PE teaching as their current role and were removed from the study, leaving one hundred and eight PE teachers eligible to complete the CPD programme. A quasi-experimental pre-post control group design for the CPD was adopted with teachers assigning themselves a start date for the CPD based on their respective work and time commitments. Teachers that could not complete the CPD by January 2022 were assigned to a wait list control group (*n* = 32) and offered the opportunity to complete the CPD once the study had finished. The remaining 76 teachers were allocated to the CPD group. All participants were asked to complete a knowledge quiz before confirming their acceptance to the CPD or wait list control group. This provided baseline MF activity knowledge data. The CPD group completed the online CPD over 10 weeks (with the understanding that three hours per week time commitment would be needed). A breakdown of the recruitment process can be seen in Figure 1. 

The first author facilitated the online CPD, corresponded with participants through online discussions, and was available through direct messaging via the CPD platform if participants experienced any problems. Participants did not use the direct messaging system for support which suggests that they encountered limited problems on the online platform during the study. Participants were allowed to progress through the CPD at their own pace, completing modules any time during the 10 weeks. Learning tasks ranged from self-directed reading, formative quizzes, and feedback opportunities. For feedback, participants were invited to comment at the end of each module on what they found useful and to reflect on their learning in an open forum to encourage group interaction and facilitate communities of practice [25]. At the end of the CPD, participants completed an exit quiz consisting of the same questions as the entry quiz. Correct answers and feedback were provided after the exit quiz. The order of these questions was randomised between participants and from the pre-CPD quiz. Once all CPD participants had completed the exit quiz, the wait list control completed their second quiz. Before CPD participants could download their certificate of completion, they received a request for feedback through a private free text box. Three free text boxes with the headings: (1) Could you please provide a brief overview of what the biggest take-home message for you was following this course? (2) What else would you like to see covered in this course? (3) Have you got any further comments regarding the course?

### 2.4. Analysis of CPD Data

The CPD course generated quantitative and qualitative data. The quantitative data were pre-and-post CPD knowledge quiz scores. Pre-and-post CPD knowledge quiz data were available from 44 participants in the CPD group (57.9% completion) and 21 in the wait list control group (65.6% completion). Twenty PE teachers did not complete the CPD despite completing the pre-knowledge quiz, with 13 of them providing a reason (more time required *n* = 6, sickness *n* = 5, pregnancy *n* = 1, change of job role *n* = 1). To address study aim 1 separate CPD knowledge change scores were generated for the CPD and wait list control groups based on pre-and-post knowledge quiz scores. These scores were first examined for normality of distribution by examination of histograms and tests for skewness. Data were not normally distributed therefore a Mann–Whitney U test was conducted to determine differences in CPD knowledge change scores between the CPD and wait list control groups. Analyses were performed using SPSS v. 27 (SPSS Inc.; Chicago, IL, USA) with statistical significance set at *p* < 0.05.

Free-text qualitative responses were pooled together and analysed thematically to address study aims 2 and 3. All open response data were managed and analysed inductively in NVivo12 (Version 12.6.0; QSR International Pty Ltd., Victoria, Australia). After the first author became familiar with the data (reading and re-reading the pooled transcription text) [34], and inductive analysis approach was taken which included producing initial codes and then searching for and reviewing themes before each final theme was clearly defined [34]. Themes were generated from the data aligned with the study aims without fitting them into a pre-existing coding frame [34]. Themes were refined in iterative steps of (1) re-reading the pooled transcription text, (2) identifying emerging codes and subsequent themes (3) refining the codes and themes with the second author [35]. The second author reviewed the coding process and provided suggestions to ensure that the coding was representative of the study aims. This process was repeated until the two authors reached a minimum 90% agreement level [36,37,38]. To present consistency of themes in the data, any themes identified and agreed upon by the first and second author that aligned with the study aims and occurred more than once were reported. Final agreed codes were summed and pen profiles were subsequently constructed to accurately illustrate the consistency of themes in the data [39]. The supplementary verbatim quotations that are provided add context to the data themes and verify participant voice. Our iterative approach to thematic analysis [34,40] and the audit trail presented above detailing and justifying the methodological decisions made throughout this aspect of the study provide transparency and trustworthiness allowing for future replication.

## 3. Results

A total of 65 teachers (55.4% male) provided complete data. The distribution of the teachers covered all four UK nations, but the majority taught in England (Wales 7.7%, Scotland 6.2%, Northern Ireland 3.1%, England 83%). Teaching experience among the teachers were relatively equally distributed across all three categories. The study characteristics of the PE teachers enrolled in the CPD and wait list control groups can be seen in Table 2. 

### 3.1. Quantitative Results

**Figure 2:**
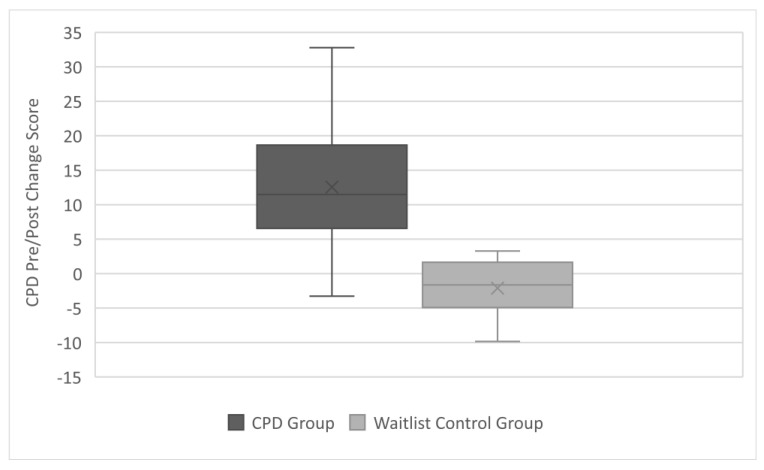
Change score median values and interquartile range between the CPD and wait list control groups.

Pre-and-post CPD knowledge quiz data were available from 65 participants (55.4% male). The median knowledge quiz change score was significantly higher in the CPD group than in the wait list control group (CPD score vs. control score; U =37, z = −5.96, *p* < 0.01). Figure 2 provides a graphical representation of change score median values and interquartile range between the CPD and wait list control groups.

### 3.2. Qualitative Results

**Figure 3:**
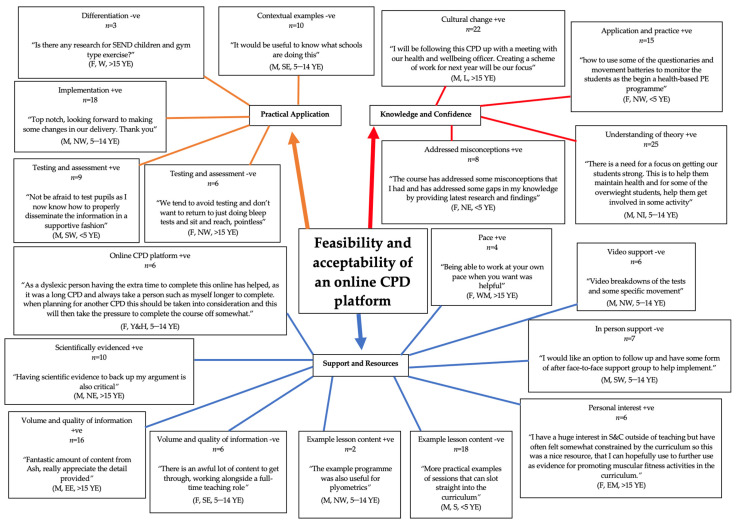
MF activity knowledge. +ve = positive; −ve = negative. M = male; F = female. SE = South East England; SW = South West England; London; NI = Northern Ireland; WM = West Midlands, England; EE = East of England; NW = North West, England; Y&H = Yorkshire and the Humber, England; S = Scotland; NE = North East and Cumbria, England; EM = East Midlands, England; W = Wales. YE = years’ experience.

To address study aims 2 and 3, negative and positive themes were identified to support the development of future online CPD courses for PE teachers. Positive (+ve) and negative (−ve) influences featured in both primary and secondary reinforcing themes. Three primary themes reflecting factors associated with the acceptability and feasibility of the online CPD were (1) practical application, (2) support and resources, and (3) knowledge and confidence (Figure 3). A further 19 secondary themes were identified: cultural change +ve (*n* = 22), application and practice +ve (*n* = 15), understanding of theory +ve (*n* = 25), addressed misconceptions +ve (*n* = 8), contextual examples −ve (*n* = 10), differentiation −ve (*n* = 3), implementation +ve (*n* = 18), testing and assessment +ve (*n* = 9), testing and assessment −ve (*n* = 6), pace +ve (*n* = 4), video support −ve (*n* = 6), in person support −ve (*n* = 7), personal interest +ve (*n* = 6), example lesson content −ve (*n* = 18), example lesson content +ve (*n* = 2), volume and quality of information +ve (*n* = 16), volume and quality of information −ve (*n* = 6), scientifically evidenced +ve (*n* = 10), online CPD platform +ve (*n* = 6). Positive (+ve) and negative (−ve) influences featured in both primary and secondary reinforcing themes.

## 4. Discussion

The aims of the present study were to (1) assess if an online CPD course can increase PE teacher knowledge of MF activity (2) assess the feasibility of an online CPD course to enhance PE teacher knowledge and competence of MF activity, and (3) assess the acceptability of the content and design of an online CPD course from PE teachers’ perspectives. Content knowledge of the PE teachers that completed the online CPD significantly increased while content knowledge scores in the wait-list control group decreased. Reported intent to change practice and integrate MF activity into lessons was evidenced amongst the PE teachers from the CPD group.

### 4.1. Knowledge and Confidence

We identified that the CPD programme resulted in increased MF activity knowledge among UK secondary school PE teachers. However, some variability was evident in change scores, with some teachers doing better than others. This may be explained by online learning processes being influenced by several internal, external and contextual factors, including time, information technology, flexibility, existing knowledge, independence and learners’ motivation and expectations [41]. Understanding these factors requires further investigation. Our findings suggested that the online CPD programme developed MF knowledge amongst the CPD participants. Furthermore, our qualitative data revealed that many PE teachers in this study responded positively to the underpinning theory regarding the health benefits of MF activity. For example:

“*This has really opened my eyes, not sure why this isn’t discussed more. I took a lot away from this and get now why strength is just as important for health as cardio fitness*”.

[Female, North West England, Over 15 years experience]

Improving content knowledge has been demonstrated to increase levels of confidence in teaching practice [42,43]. Our findings are consistent with previous research and suggest an intention to translate acquired knowledge into practice with a view to drive cultural change within PE teachers’ respective schools. This is a positive finding given the paucity of pedagogical research into the delivery of MF activity in schools and supports the acceptability of online CPD amongst study participants. However, as evidenced in the present study, such success is only achieved if the content delivered is underpinned by empirical evidence and informed by the needs of the teacher [25]. The theoretical aspects of the online CPD programme and the relevance of the content to teachers’ interests may explain the intent to apply newly acquired knowledge into practice. This requirement has been documented elsewhere in the literature and has been suggested to elicit greater CPD engagement and adherence [22,44]. Collectively, these findings further support the need for CPD that is content knowledge centric and underpinned by up-to-date research.

PE teachers are required to be highly qualified in the content of the subject area in which they teach (i.e., high levels of content knowledge) [44]. However, expertise in content alone is inadequate and an understanding of pedagogical content knowledge is required to successfully plan and implement MF activity that aligns with individual student learning styles and needs [44,45]. Due to a lack of MF activity research and practice in schools [29], there was limited school-based pedagogical knowledge research to underpin the CPD content. However, when provided with content knowledge alone, our findings suggested that PE teachers could adapt content knowledge to their existing pedagogical knowledge. To overcome the paucity of pedagogical research in MF activity, this CPD relied on existing frameworks to deliver MF activity that have primarily been developed for athletic development processes. The models used as frameworks in this CPD were long-term athletic development models that focussed on specific modes of MF activity [46]. There was an emphasis on plyometric movements in the form of the plyometric progression model to align the content with the needs and requirements identified in the CPD development process [47]. Furthermore, the models were presented alongside a theoretical periodisation plan that was aligned to a secondary school term to provide insight into how MF activity could be implemented in schools [13]. Despite the lack of pedagogical knowledge specific to MF activity, PE teachers in this study found the athletic models presented useful to add context to implementation. For example:

“*The athletic models were useful and something we can use for our lessons. They will feature in our GCSE class next year to help pupils understand periodisation and we can use them to plan our lesson progressions*”.

[Male, London, 5–14 years experience]

Our findings are supportive of providing PE teachers with content knowledge they can translate into practice based upon their existing pedagogical knowledge. Therefore, future MF CPD can utilise a similar authentic pedagogical approach to PE teacher learning. Authentic pedagogy is a systematic learning strategy which helps the learners (PE teachers) to develop solutions in real-world problems guided by proper instructional approaches [48]. Such approaches may allow CPD providers to focus on up-to-date content knowledge whereby there may be a dearth of existing literature on pedagogical approaches to new developments. 

### 4.2. Practical Application

PE teachers in this study suggested they would make changes to their PE provision to include MF activity following the online CPD. Previous work has suggested that a lack of interaction with CPD facilitators may hinder practical application of learning [25,49]. Our findings may contrast with previous literature following the co-production of the CPD programme with current teachers. The online CPD accounted for suggestions provided by PE teachers during the CPD development and included plyometric movements such as jumping and bounding to allow schools with minimal equipment to put learning into practice. Such movements are underpinned by existing literature as being effective school-based MF activities [11] and are movements most teachers are familiar with. This ensured MF activity was coherent with other aspects of the PE curriculum and may explain the intent to integrate MF activity into practice alongside existing pedagogical knowledge [27,50]. For example:

“*I already knew about plyometric exercise through my UKA (UK Athletics) course. The CPD helped take what I know and put it into a gym lesson. It makes sense to use the plyometric progression model and help pupils work towards their own plan and fitness, something they can even use for GCSE work. Thank you*”.

[Male, North East England, over 15 years experience]

However, our findings suggested that a lack of contextual examples from other schools that are delivering MF activity. Previous work has highlighted that teachers will need to see evidence of initiatives resulting in improved pupil outcomes before changing attitudes and beliefs prior to implementing new learning [50]. This is a concern regarding the fidelity of MF activity in schools given the lack of contextual examples to refer to and may lead to hesitancy in implementing MF activity among PE teachers. Our findings suggested that PE teachers require contextual examples during CPD. For example:

“*Who else is doing this (MF activity)? It would be good to see how others have used it (MF activity) in their PE lessons*”.

[Female, East of England, under 5 years experience]

Our findings demonstrated that future online MF CPD courses may benefit from providing contextual examples from other schools to further support the translation of CPD content into practice. One approach may be to develop Professional Learning Communities. Professional Learning Communities provide an opportunity for collective learning and application and, shared individual practice [51], which are suggested to improve practical application of acquired knowledge. A benefit to the Professional Learning Community structure for MF activity CPD is the top-down structure and obligatory involvement [52]. This may break the cycle of teachers engaging in CPD that aligns with their interests and not of their developmental requirements [53]. However, this approach would require further collaboration with school policy makers to overcome barriers that preclude such initiatives and ensure policy makers understand the benefits of MF activity.

PE teachers in this study expressed that they were more likely to conduct tests and assessments of MF to inform their MF activity lessons when provided with a rationale and standardised protocols. The efficacy of MF activity is underpinned by appropriate testing and assessment to progress individuals safely [11]. Therefore, developing an understanding of testing and assessments of MF may lead to increases in the quantity and quality of MF activity and provide a non-invasive objective assessment of the impact of school-based MF interventions [15,54]. However, in schools, tests and assessments in PE have been highly contested [15,55] and some PE teachers in this study were hesitant to implement testing and assessments due to preconceived biases surrounding broader testing beyond MF. PE teacher concerns centred around the value of testing and assessment in other areas of PE such as the bleep test for cardiorespiratory fitness and suggests more work is needed in this area. Despite our findings suggesting an intent to conduct MF activity, it is difficult to conclude this will translate into action. Future research should jointly monitor student outcomes and teacher learning following CPD to establish a link between acquired knowledge and the impact this has on student outcomes of MF.

### 4.3. Support and Resources

One of the challenges of online CPD is the provision of meaningful support [56]. PE teachers have reported a lack of time to remain current with research developments and report feelings of isolation [50]. To ensure the online CPD allowed for teachers to ascribe their own meaning and understanding of the newly acquired MF activity knowledge, collaborative engagement was encouraged in this study through discussion points during each module [53]. This process encouraged participants to be active learners and reflect on how the delivered content knowledge could be adapted to suit their respective pedagogical practices in PE [27,57]. Unfortunately, the discussion opportunities did not generate meaningful discussion around the application and pedagogical approaches to delivering MF activity, with teachers requesting further support and clarity on lesson delivery in schools. Future CPD may benefit from co-delivery, utilising a PE teacher experienced in MF activity delivery alongside a university's research-based knowledge and expertise to drive discussion. 

Teachers who are satisfied with CPD content are likelier to adopt and implement programs as intended [44]. Therefore, this study ensured the online CPD met PE teacher needs and interests through the co-production of CPD content. This was evidenced in our findings whereby participants suggested they had a personal interest in the content and appreciated the scientific underpinning in which it was delivered. This aligns with suggestions made in existing literature that accounting for personal needs and interests is a key component for CPD success [53]. These findings demonstrate that although concerns regarding online CPD lacking nuance and individualisation exist [22,58], teacher interests and needs can be met through online CPD. This may be explained by the co-production of the CPD and highlights the need for future research to adopt a similar approach. Another explanation for the positive CPD experience could be attributed to the course delivered by a trusted individual working within a university. It has been suggested that the delivery of CPD by an individual or organisation with appropriate expertise may increase the likelihood of CPD content resonating with the teacher [50]. This is supportive of PE teachers reporting links with universities providing stimulating and interesting interactions that extend beyond their routine teaching [50] and suggests that similar higher education and PE teacher relationships should be sought.

A key strength of the online delivery was extending content delivery beyond one day, allowing for a greater amount of content to be digested over a number of weeks [59]. Moreover, the extended and flexible training allowed the pace of delivery to suit a range of individuals, particularly individuals who wanted to review and revisit content to either cement learning or assist in the learning process. For example:

“*Going at my own pace in my own time was great. I could work it around work and home life. I have struggled with workshops in the past, especially with having issues around my learning ability. I have to go over things and take my time*”.

[Female, Yorkshire and the Humber, 5–14 years experience]

Extending CPD across 10 weeks allowed for a sustained approach typically defined as extending beyond one day [50]. This extended approach may help support MF activity implementation [44]. However, our findings also highlighted that although PE teacher content knowledge increased, there was still a requirement to be provided with lesson plan templates. A lack of example lesson content in this study may impact MF activity implementation. Future online CPD content should ensure downloadable lesson content is provided to help support lesson or activity implementation and increase the feasibility of MF activity CPD. 

### 4.4. Limitations

Limitations of this study relate to the imbalanced sample between the four UK home countries, and the risk of self-selection bias. However, steps were taken to limit such bias, including co-production of the CPD content and the application to gain CPD point recognition. Furthermore, findings from this study should be interpreted with caution as the cross-sectional design cannot claim causality. The sample size for the quantitative analysis was modest and as such, statistical power may have been lacking. However, the mixed methods approach to data collection allowed for added context to support the quantitative data.

## 5. Conclusions

This is the first study in the UK to investigate the feasibility and acceptability of an online MF activity CPD programme. Overall, MF activity knowledge of the PE teachers that completed the online CPD increased while it decreased in the control group. Evidence that an online CPD programme results in increased MF activity may help overcome the reported barriers to MF activity implementation in schools and provides a feasible way to deliver MF activity CPD to PE teachers. Furthermore, PE teachers that completed the CPD reported the online platform was beneficial to overcoming the limitations of face-to-face CPD such as time and financial constraints and suggested the content covered was beneficial and appropriate to their teaching. Whilst the online CPD was on acceptable method of delivering CPD, the benefit to co-production should not be overlooked. This approach allowed the CPD to align to teachers’ requirements and future research should adopt a similar approach. Finally, future work is required to establish links between teachers’ learning following CPD, the translation into PE practice and student MF outcomes.

## Figures and Tables

**Table 1:** Summary of CPD modules.

Module	Overview
**Preliminary Stage**	Welcome video, participant information, consent, and the entry knowledge quiz.
**Introduction**	Brief rationale, definitions of key terminology and a group introduction forum.
**Physical Activity**	Overview of PA guidelines, health benefits of MF, addressing common misconceptions surrounding MF activity and two discussion forums about MF.
**Muscular Fitness Development**	Implementation of MF in schools, programming and periodization, common misconceptions.
**Plyometrics**	Introduction to plyometrics, safety considerations, programming, plyometric progression model, plyometric discussion forum.
**Delivery and Long-Term Development**	Teacher conduct in delivering MF activity, delivery and class management considerations, physical development models, discussion forum on MF delivery.
**Monitoring and Assessment**	Assessing MF, overview of common MF assessment tools, conducting MF assessments, discussion forum for MF assessments.
**Round Up**	CPD round up, knowledge quiz, exit survey.

**Table 2:** Characteristics of PE teachers enrolled in CPD and wait list control groups.

Characteristics	All (*n* = 65)	CPD Group (*n* = 44)	Wait List Control Group (*n* = 21)
**Sex % (*n*)**			
**Male**	55.4 (36)	56.8 (25)	52.4 (11)
**Female**	44.6 (29)	43.2 (19)	47.6 (10)
**School Location % (*n*)**			
**Cymru Wales**	7.7 (5)	9.1 (4)	4.8 (1)
**East of England**	7.7 (5)	4.5 (2)	14.3 (3)
**North East and Cumbria**	3.1 (2)	-	9.5 (2)
**East Midlands**	13.8 (9)	15.9 (7)	9.5 (2)
**London**	7.7 (5)	9.1 (4)	4.8 (1)
**North West**	10.8 (7)	11.4 (5)	9.5 (2)
**Northern Ireland**	3.1 (2)	2.3 (1)	4.8 (1)
**Scotland**	6.2 (4)	4.5 (2)	9.5 (2)
**South East**	15.4 (10)	18.2 (8)	9.5 (2)
**South West**	7.7 (5)	6.8 (3)	9.5 (2)
**West Midlands**	10.8 (7)	11.4 (5)	9.5 (2)
**Yorkshire and the Humber**	6.2 (4)	6.8 (3)	4.8 (1)
**Teaching Experience (years) % (*n*)**			
**<5**	35.4 (23)	36.4 (16)	33.3 (7)
**5–14**	33.8 (22)	34.1 (15)	33.3 (7)
**>15**	30.8 (20)	29.5 (13)	33.3 (7)

## Data Availability

Supporting data for this study is not openly available as participants did not provide informed consent for data sharing.
